# Ascorbic acid insufficiency impairs spatial memory formation in juvenile AKR1A-knockout mice

**DOI:** 10.3164/jcbn.19-41

**Published:** 2019-09-11

**Authors:** Kazuki Kurihara, Takujiro Homma, Sho Kobayashi, Mototada Shichiri, Hiroki Fujiwara, Satoshi Fujii, Ken-ichi Yamada, Masaki Nakane, Kaneyuki Kawamae, Junichi Fujii

**Affiliations:** 1 Department of Biochemistry and Molecular Biology, Graduate School of Medical Science, Yamagata University, 2-2-2 Iidanishi, Yamagata 990-9585, Japan; 2 Department of Anesthesiology, Faculty of Medicine, Yamagata University, 2-2-2 Iidanishi, Yamagata 990-9585, Japan; 3 Biomedical Research Institute, National Institute of Advanced Industrial Science and Technology (AIST), 1-8-31 Midorigaoka, Ikeda, Osaka 563-8577, Japan; 4 Department of Physiology, Faculty of Medicine, Yamagata University, 2-2-2 Iidanishi, Yamagata 990-9585, Japan; 5 Physical Chemistry for Life Science Laboratory, Faculty of Pharmaceutical Sciences, Kyushu University, 3-1-1 Maidashi, Higashi-ku, Fukuoka 812-8582, Japan; 6 AMED-CREST, Japan Agency for Medical Research and Development, 1-7-1 Otemachi, Chiyoda-ku, Tokyo, Japan; 7 Department of Emergency and Critical Care Medicine, Faculty of Medicine, Yamagata University, 2-2-2 Iidanishi, Yamagata 990-9585, Japan

**Keywords:** ascorbic acid, AKR1A, Morris water maze, spatial memory

## Abstract

AKR1A, an aldo-keto reductase, is involved in the synthesis of ascorbic acid as well as the reduction of a variety of aldehyde compounds. AKR1A^−/−^ mice produce considerably less ascorbic acid (about 10%) compared to AKR1A^+/+^ mice and require ascorbic acid supplementation in order to breed. To elucidate the roles played by AKR1A in spatial memory, AKR1A^−/−^ male mice were weaned at 4 weeks of age and groups that received ascorbic acid supplementation and no supplementation were subjected to a Morris water maze test. Juvenile AKR1A^−/−^ mice that received no supplementation showed impaired spatial memory formation, even though about 70% of the ascorbic acid remained in the brains of the AKR1A^−/−^ mice at day 7 after weaning. To the contrary, the young adult AKR1A^−/−^ mice at 13–15 weeks of age maintained only 15% of ascorbic acid but showed no significant difference in the spatial memory compared with the AKR1A^+/+^ mice or ascorbic acid-supplemented AKR1A^−/−^ mice. It is conceivable that juvenile mice require more ascorbic acid for the appropriate level of formation of spatial memory and that maturation of the neural system renders the memory forming process less sensitive to an ascorbic acid insufficiency.

## Introduction

Ascorbic acid (AsA), vitamin C, has pleiotropic roles in maintaining healthy conditions in the mammalian body.^(1)^ A deficiency of AsA results in metabolic abnormalities and an imbalance of redox homeostasis, notably in the cardiovascular system, and in severe cases, fatality increases, as typically observed in scurvy. AsA is concentrated in central nervous system (CNS) and appears to be involved in a number of metabolic processes including dopamine β-hydroxylase activity in the biosynthesis of catecholamine.^(2)^ AsA is released from astrocytes upon stimulation by glutamate and is then taken up by neurons.^(3)^ While AsA exerts neuroprotective action by suppressing oxidative stress that is triggered by glutamate excitotoxicity,^(4)^ it also promotes oligodendrocyte generation and remyelination. These results imply that AsA could have therapeutic potential for the treatment of demyelinating diseases, such as Multiple Sclerosis.^(5)^ Thus AsA is beneficial in the CNS from standpoint of physiology and pathology.

Rodents are popular laboratory animals, but because they have the ability to synthesize AsA they cannot be used as model animals in investigations of the AsA functions. The enzyme l-gulono-γ-lactone oxidase (GULO) catalyzes the final step in the biosynthesis of AsA synthesis, using molecular oxygen and releases AsA.^(6)^ Primates are incapable of synthesizing AsA due to a mutation in the GULO gene, which likely occurred about 63,000,000 years ago.^(7)^ The genetic ablation of GULO shows a total defect in AsA synthesis in mice.^(8)^ In turn, gluconolactonase (GNL) catalyzes the dehydration of l-gulonate to l-gulono-γ-lactone, the penultimate reaction in the AsA synthesis pathway. Mice with a genetic ablation of GNL, which is identical to the senescence marker protein 30, also show complete inability to biosynthesize AsA.^(9)^

Aldehyde reductase (AKR1A) and aldose reductase (AKR1B), members of the aldo-keto reductase (AKR) superfamily,^(10)^ have been reported to catalyze the NADPH-dependent reduction of d-glucuronic acid to l-gulonate, the reaction immediately before that catalyzed by GNL.^(11,12)^ The ablation of these genes, therefore, result in impaired AsA synthesis, and the contributions of AKR1A and AKR1B to AsA synthesis in mice reduced to 85–90% and 10–15%, respectively. While AKR1A^−/−^ mice show pathological characteristics similar to scurvy and do not survive beyond one year in the absence of AsA supplementation,^(13)^ neither the phenotypic abnormality associated with an AsA insufficiency nor an altered longevity has been reported for AKR1B^−/−^ mice.^(11,14,15)^ AKR1A also plays roles in metabolic reactions in addition to AsA synthesis, which appears to confirm its existence in primates.^(16–19)^

Regarding the function of AsA in CNS, AKR1A^−/−^ mice are hypersensitive to pentobarbital anesthesia and this can be reversed by the administration of AsA.^(20)^ On the other hand, AsA has no influence over the aggressive behavior caused by an AKR1A deficiency.^(21)^ Thus, AKR1A appears to play differential roles in the CNS or other neuronal systems, but no rational explanation has been provided for this. In this study we subjected AKR1A^−/−^ mice to the Morris water maze test and evaluated the effects of an AsA insufficiency in the light of the spatial memory formation.

## Materials and Methods

### Animals

AKR1A^−/−^ mice with a C57BL/6 background that were generated using a gene-targeting technique,^(12)^ were bred in our institution and used throughout the study. The male AKR1A^+/+^ and AKR1A^−/−^ mice were weaned at 30 days of age and fed a standard diet (Picolab 5053, LabDiet, St. Louis, MO) *ad libitum* with free access to either water or water containing 1.5 mg/ml AsA until they were used. The supplemented AsA concentration was sufficient to allow the AKR1A^−/−^ mice survive longer than one year.^(13)^ Animal experiments were performed in accordance with the Declaration of Helsinki under the protocol approved by the Animal Research Committee at our institution.

### Morris water maze test

To evaluate spatial memory in the AKR1A^−/−^ mice, the Morris water maze test was performed.^(22)^ A circular target platform (10 cm in diameter) was immersed in a pool (diameter 120 cm) 7 cm below the surface of the water, and four black-and-white drawings were attached to the inside wall of the pool above the water surface. The water temperature was maintained at 20 ± 1°C. The test was conducted on 4 consecutive days. Each mouse was examined four times per day, starting at a different position each time, in submerged platform trials in white-colored water containing skim milk. The swimming was video-tracked for 90 s. When the mouse reached the platform within 90 s, it was allowed to remain on the platform for 15 s and view the drawings. If the mouse did not reach the platform within 90 s, it was forced to view the drawing on the platform for 15 s. Escape latency, escape distance and swimming speed were measured in the quadrant where the platform was located using a video tracking system Compact VAS ver 3.0x (Muromachi Kikai, Tokyo, Japan).

### Measurement of the reduced form of AsA

A fluorescent probe, 15-(Naphthalen-1-ylamino)-7-aza-3, 11-dioxadispiro[5.1.58.36]hexadecan-7-oxyl (Naph-DiPy), was synthesized^(23)^ and used to measure the concentration of AsA.^(18)^ Fresh blood plasma prepared from either the tail vein or the heart at autopsy was used for the AsA assay. In a typical run, a blood sample was collected in the presence of excess EDTA. The blood plasma was obtained by centrifugation of the sample at 800 × *g* for 3 min at room temperature. Hippocampus tissue was dissected from mice, quickly frozen in liquid nitrogen, and stored at −80°C until used. After homogenizing the hippocampus tissue in 10 volumes of phosphate-buffered saline followed by centrifugation at 17,400 × *g* for 15 min at 4°C, the supernatant was diluted with phosphate-buffered saline. The blood plasma or the diluted tissue extract were incubated with Naph-DiPy for 30 min at room temperature in the dark. The AsA concentration was calculated by measuring the fluorescence at an excitation wavelength of 310 nm and an emission wavelength of 430 nm using a microplate reader (Valioskan Flash, Thermo Fisher Scientific, Waltham, MA).

### Measurement of choline, acetylcholine, glutathione and cysteine

LC-MS analyses of choline, acetylcholine, cysteine (Cys), and glutathione (GSH) in hippocampus extracts were performed as described in a previous report^(24)^ with minor modifications.^(25)^ 10 mg of tissue samples were homogenized in 100 µl buffer containing 20 mM *N*-ethylmaleimide (NEM) and 50 mM ammonium bicarbonate, pH 8.0, to block the sulfhydryl groups in Cys and GSH. The resulting homogenate was incubated for 10 min at room temperature. After adding a 200 µl portion of methanol containing 5 µM *N*-methylmaleimide (NMM)-derivatized GSH as an internal standard and another 200 µl of chloroform, the mixture was thoroughly stirred and centrifuged at 12,000 × *g* for 15 min 4°C. The upper aqueous layer was filtered through a 0.45 µm filter (Millex^®^-LH, Merck Millipore, Burlington, MA). A 90 µl aliquot of the filtrate was lyophilized, the residue dissolved in 30 µl of 50% acetonitrile, and subjected to liquid chromatography (LC)-mass spectrometry (MS) analysis. A Q Exactive Hybrid Quadruple-Orbitrap mass spectrometer (Thermo Fisher Scientific) equipped with a heated electrospray ionization source was operated in the positive ionization mode for this analysis. An Ultimate 3000 liquid chromatography system consisted of a WPS-3000 TRS autosampler, a TCC-3000 RS column oven, and a HPG-3400RS quaternary pump (Dionex, Sunnyvale, CA). A SeQuant^®^ ZIC^®^-pHILIC column (2.1 × 150 mm, 5 µm particle size; Merck KGaA, Germany) was maintained at 30°C. The mobile phase A was 20 mM ammonium bicarbonate, pH 9.8, and the mobile phase B was 100% acetonitrile. System control, data acquisition and quantitative analysis were performed with the Xcalibur 2.2 software. Standard curves for choline, acetylcholine, GSH-NEM, and Cys-NEM showed linearity in concentration ranges examined.

### Measurement of neuroactive amines in hippocampus tissue

The levels of amines were measured by high performance liquid chromatography (HPLC), as previously described.^(26)^ Hippocampus tissue was homogenized in 0.2 M perchloric acid (10 µl/mg tissue) containing 100 µM EDTA-2Na and isoproterenol (100 ng) was added as an internal standard. After centrifugation at 20,000 × *g* for 15 min, the supernatants were transferred to another tube and the pH adjusted to 3 by adding 1 M sodium acetate. Samples were diluted to 1/20 and 1/400 with 0.2 M perchloric acid containing 100 µM EDTA-2Na. Samples (10 µl) were then analyzed by HPLC.

### Histological analyses of brain

Histological analyses were performed at the Pathological Analysis Center, Institute for Promotion of Medical Science Research, Yamagata University. Dissected brains were fixed in 10% buffered formalin followed by embedding in paraffin. Brain sections (5 µm thick) were subjected to either hematoxylin and eosin (H&E) staining or Nissl staining. Photographs of the sections were taken with a BZ-X700 microscope (Keyence, Osaka, Japan).

### Protein preparation

Brains were weighed and homogenized in 5 volumes of RIPA buffer (25 mM Tris-HCl pH 7.5, 150 mM NaCl, 1% NP-40, 1% sodium deoxycholate, 0.1% SDS) containing a protease inhibitor cocktail (Roche) and centrifuged at 17,400 × *g* in a microcentrifuge. The supernatant was used for protein determination using a Pierce^®^ BCA^TM^ Protein Assay Kit (Thermo Fisher Scientific).

### Immunoblot analyses

Aliquots of protein were separated on 10% or 15% SDS-polyacrylamide gels and electroblotted onto polyvinylidene difluoride membranes (GE Healthcare, Chicago, IL). The blots were blocked with 3% skim milk in tris-buffered saline-containing 0.1% Tween-20 (TBST), and were then incubated with the antibodies. Some of the primary antibodies used in this study had been produced in a previous study; AKR1A,^(17)^ AKR1B,^(27)^ SOD1,^(28)^ SOD2,^(28)^ GPX1,^(29)^ and catalase (Merck Millipore, 219010), and β-actin (Santa Cruz Biotechnology, sc-69879, Dallas, TX). Horseradish peroxidase (HRP)-conjugated goat anti-rabbit or anti-mouse IgG antibody (Santa Cruz Biotechnology) were used as secondary antibodies. After washing, the presence of bound HRP was detected by measuring the chemiluminescence using Immobilon western chemiluminescent HRP substrate (Merck Millipore) on an image analyzer (ImageQuant LAS500, GE Healthcare).

### Statistical analysis

The results are expressed as the mean ± SEM. Statistical analysis was performed using the Student *t* test or one-way ANOVA, followed by the Tukey-Kramer test for multiple groups. A *p* value of less than 0.05 was considered significant. *p<0.05, **p<0.01, ***p<0.001.

## Results

### AKR1A knockout impairs spatial memory formation in juvenile mice but not young adult mice

**Fig. 1:**
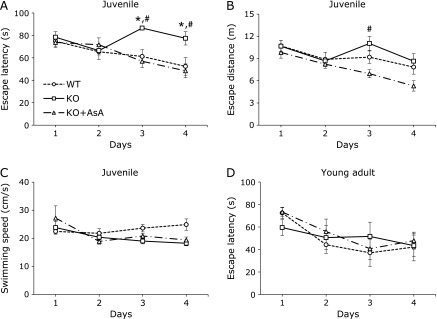
Morris water maze acquisition performance in the mice. Spatial learning was evaluated by the Morris water maze task on juvenile (A–C, *n* = 10–12) and young adult mice (D, *n* = 6 or 7). Identical sequences of start locations were used each day. Data are latency (s) to reach the goal (A and D), escape distance (B), and swimming speed (C). Values are expressed as the mean ± SEM. *p<0.05, compared with the AKR1A^+/+^ mice. ^#^*p*<0.05, compared with the AKR1A^−/−^ mice supplemented with AsA. WT, AKR1A^+/+^ mice; KO, AKR1A^−/−^ mice; KO + AsA, AKR1A^−/−^ mice supplemented with AsA.

During their period of lactation, the drinking water for the AKR1A^−/−^ mice was supplemented with AsA (1.5 g/L) for the purpose of breeding but was ceased at the time of weaning at 30 days after birth, while a second group of AKR1A^−/−^ mice continued to receive the AsA supplement to distinguish the effects of AsA from other functions of AKR1A. We subjected three groups of juvenile male mice at 4-weeks of age; AKR1A^+/+^ mice, AKR1A^−/−^ mice without AsA supplementation, and AKR1A^−/−^ mice with AsA supplementation, to the Morris water maze test after weaning. The findings indicated that a latency to reach the platform was significantly decreased in the AKR1A^+/+^ mice and the AKR1A^−/−^ mice with AsA supplementation during the trial period (Fig. 1A). On day 1 and day 2, no statistically significant differences were noted among the three groups, but on day 3 and day 4, the AKR1A^−/−^ mice without AsA supplementation showed a delay in escape latency compared with the other groups. The escape distance was not significantly shortened in the AKR1A^−/−^ mice during the session but gradually became shortened in the case of the AKR1A^+/+^ mice or the AKR1A^−/−^ mice with AsA supplementation (Fig. 1B). Swimming speed remained about the same during the session for all three groups (Fig. 1C). On the other hand, when the same experiment was conducted on young adult mice at 13–15-weeks of age, no significant differences were observed in latency among three groups of mice (Fig. 1D). Body weights were about the same among the three groups of mice (data not shown).

### AsA levels are preserved in the brain longer than other organs

**Fig. 2:**
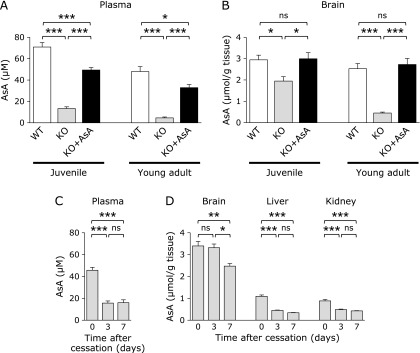
Changes in AsA levels in organs. AsA levels were measured by a fluorometric assay using Naph-DiPy as a fluorescent probe. AsA levels in blood plasma (A) and brains (B) tissues in juvenile mice (*n* = 8 or 9) and young adult mice (*n* = 4 or 5). Changes in AsA levels in blood plasma (C) and brain, liver and kidney tissues (D) at day 0, 3, and 7 after cessation of AsA supplementation to juvenile AKR1A^−/−^ mice (*n* = 4). Values are expressed as the mean ± SEM. *p<0.05, **p<0.01, ***p<0.001. WT, AKR1A^+/+^ mice; KO, AKR1A^−/−^ mice; KO + AsA, AKR1A^−/−^ mice supplemented with AsA.

We measured AsA levels in blood plasma and whole brains of juvenile mice at 5 weeks of age and young adult mice at 13–14 weeks of age. The plasma levels of AsA were about 15% in juvenile AKR1A^−/−^ mice and less than 10% in young adult AKR1A^−/−^ mice compared to corresponding AKR1A^+/+^ mice (Fig. 2A). Supplementation with AsA increased these levels to 70% of the corresponding values for AKR1A^+/+^ mice in juvenile AKR1A^−/−^ mice and young adult AKR1A^−/−^ mice, respectively. To the contrary, the brain maintained higher levels of AsA; 70% in juvenile AKR1A^−/−^ mice and 15% in young adult AKR1A^−/−^ mice (Fig. 2B). It is also noteworthy that the AKR1A^−/−^ mice that received AsA-supplementation maintained the same levels of AsA as that for the AKR1A^+/+^ mice.

When AsA levels were measured in other organs, the liver and kidney, as well as blood plasma and brain of juvenile AKR1A^−/−^ mice at days 0, 3 and 7 after the cessation of AsA at 4 weeks of age, the levels declined rapidly in blood plasma, liver, and kidney (Fig. 2C and D). However, the brains of AKR1A^−/−^ mice originally contained several-fold higher levels of AsA than the liver or kidney and more than 70% of the AsA was preserved on day 7. These findings suggest that there is a specific mechanism for preserving AsA in the brain compared to other organs.

### Levels of neurotransmitters amines, acetylcholine, redox compounds in the hippocampus

We hypothesized that the production of neurotransmitters might be affected by the status of AsA and that this might influence the neuronal function of the juvenile mice. Because dopamine is released into the dorsal hippocampus, binds to D1/D5 receptors and promotes several responses, including spatial learning,^(30)^ the dopamine levels, together with other neurotransmitter amines in the hippocampus were measured by HPLC. Essentially no significant differences were observed in their levels among three mice groups, except for finding that the norepinephrines were slightly increased in AKR1A^−/−^ mice with AsA supplementation compared with AKR1A^+/+^ mice (Table 1). Since the hippocampus is regarded as the site of action for the effects of nicotine on spatial learning,^(31)^ we also measured the levels of acetylcholine and choline together with the antioxidative molecules, cysteine and GSH, in hippocampus area of three groups of juvenile mice by LC-MS. Again, there were no significant differences in their levels among the three groups of the mice.

### No changes in brain histology or related proteins

**Fig. 3:**
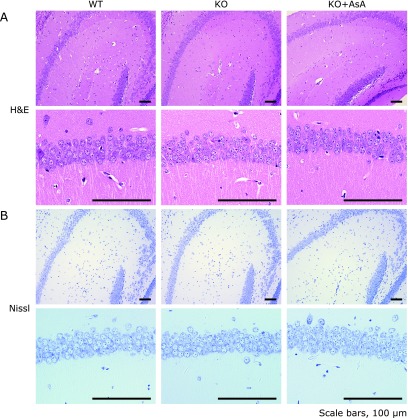
Histological analyses of hippocampus tissue of the mice. H&E (A) and Nissl staining (B) were performed on hippocampus sections of juvenile mice. Representative images were shown on the sections from three groups of the mice at 4 weeks of age (*n* = 2). WT, AKR1A^+/+^ mice; KO, AKR1A^−/−^ mice; KO + AsA, AKR1A^−/−^ mice supplemented with AsA.

**Fig. 4:**
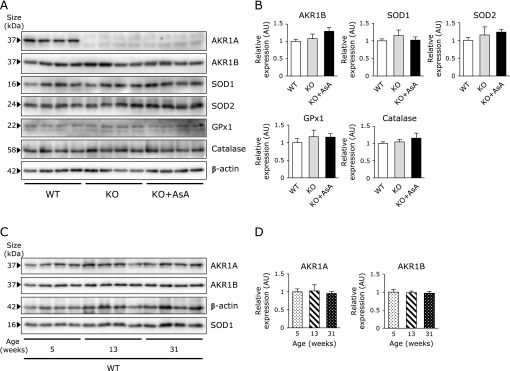
Protein levels of AKR1A, AKR1B, and antioxidative enzymes. (A) Proteins were extracted from brains of the juvenile AKR1A^+/+^ mice and AKR1A^−/−^ mice with or without AsA supplementation. Immunoblot analyses of AKR1A, AKR1B, enzymes involved in antioxidation (SOD1, SOD2, GPX1, and catalase), and β-actin were performed. (B) Immune reactive bands were semi-quantified by scanning the blot membranes and normalized to the corresponding β-actin levels. Data are expressed as the mean ± SEM. (C) Proteins were extracted from brains of the AKR1A^+/+^ mice at 5, 13, and 31 weeks-old mice. Immunoblot analysis of AKR1A, AKR1B, SOD1 and β-actin were performed. *n* = 4. (D) Immune reactive bands were semi-quantified by scanning the blot membranes and normalized to the corresponding SOD1 because β-actin levels were found to increase during aging. Values are expressed as the mean ± SEM. No statistical differences were observed among three groups. WT, AKR1A^+/+^ mice; KO, AKR1A^−/−^ mice; KO + AsA, AKR1A^−/−^ mice supplemented with AsA.

When histological analyses of the brain were performed on the three groups of mice at 5 weeks of age, no evident changes were observed in the brain sections that had been subjected to either H&E staining (Fig. 3A) or Nissl staining (Fig. 3B). To further explore the reason for the defect in the spatial memory formation in the AKR1A^−/−^ mice, the levels of AKR1A and AKR1B and antioxidative enzymes in the brains of these mice were assessed by immunoblot analysis. The absence of AKR1A was confirmed in the AKR1A^−/−^ mice, and no changes were observed in the levels of AKR1B among the three groups of mice (Fig. 4A and B). The levels of major antioxidative enzymes, SOD1, SOD2, GPX1, and catalase remained unchanged. We also assessed possible variations in AKR1A and AKR1B levels in the AKR1A^+/+^ mice during aging (Fig. 4C and D) and no changes in their levels were detected at 5, 13, and 31 weeks of age. Thus the AKR enzymes and antioxidative system remained unchanged during aging and appeared to not be the cause for the differential response in the neuronal function.

### Isoflurane anesthesia had no affect on the spatial memory formation in young adult mice

**Fig. 5:**
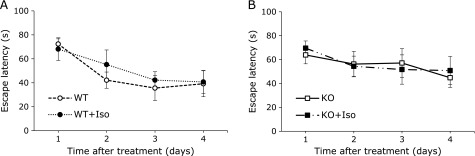
Effects of isoflurane on spatial memory formation. Young adult AKR1A^+/+^ mice (WT + Iso; A) and AKR1A^−/−^ mice (KO + Iso; B) were treated with 2% isoflurane (Iso) for 2 h on day 0 and then subjected to Morris water maze test. Identical sequences of start locations were used each day. Data are latency (s) to reach the goal (*n* = 7 for each group). Data corresponding to the AKR1A^+/+^ and AKR1A^−/−^ control mice were redrawn from the corresponding data in Fig. 1D for reference. Values are expressed as the mean ± SEM. No statistical difference was observed between control group and isoflurane-treated group in both genetic mice.

Because a variety of stress conditions can exerts an oxidative insult and AsA suppresses it by virtue of its antioxidative function, it is possible that stress caused by anesthesia might affect the spatial memory formation in the AsA-insufficient adult AKR1A^−/−^ mice. We used isoflurane, an inhaled anesthetic, that reportedly affects spatial memory formation via oxidative stress or endoplasmic reticulum stress in juvenile rodents.^(32,33)^ Male AKR1A^+/+^ mice or AKR1A^−/−^ mice that had grown to 12–13 weeks of age without AsA supplementation were treated with a relatively high dose of isoflurane (2%) for 2 h on day 0, and were then subjected to the water maze test in following days as shown in Fig. 1 (Fig. 5). The results indicated that the isoflurane treatment had no significant effects on spatial memory formation both in the AKR1A^+/+^ mice or in the AKR1A^−/−^ mice, suggesting that the young adult mice had a robust resistance against an AsA insufficiency.

## Discussion

The findings reported herein show that AKR1A^−/−^ mice at the juvenile stage had a defect in spatial memory formation, as judged by the results of a water maze test (Fig. 1A). Although AsA levels were relatively well preserved in brains compared to other organs after the cessation of supplementation (Fig. 2D), spatial memory formation was impaired by a minor decline in the AsA contents in juvenile AKR1A^−/−^ mice. The impairment in the memory formation became less evident in adult AKR1A^−/−^ mice (Fig. 1D), implying that the maturation of the neuronal system rendered the CNS resistance to an AsA deficiency.

Roles of AsA in CNS have been extensively examined in GULO^−/−^ mice^(34,35)^ and GNL^−/−^ mice.^(9,36,37)^ However, there are substantial differences between our results and these studies. The ablation of AKR1A continues to enable the production AsA at a level of 10–15%,^(11,12)^ while the ablation of either GULO or GNL resulted in a decrease in AsA production to negligible levels.^(8,9)^ The complete absence of AsA for a long period would affect a variety of physiologic processes, and would show more profound phenotypic abnormalities in GULO^−/−^ or GNL^−/−^ mice than in the AKR1A^−/−^ mice. The advantage of using AKR1A^−/−^ mice would be that a certain amount of AsA would persist in the brain, which is similar to the actual pathological state of a human with an insufficient AsA dietary intake.

High levels of AsA were retained in the brains of these mice, while the AsA contents rapidly declined in other organs of the AKR1A^−/−^ mice after the cessation of AsA supplementation (Fig. 2), which is consistent with the previous reports using other genetically modified mice.^(34)^ An AsA deficiency during gestation causes developmental defects in the neonatal cerebellum and impairs its function in adult GULO^−/−^ mice via alteration in cellular composition at that location.^(35)^ Impaired spatial memory has also been reported in juvenile guinea pigs,^(38)^ which, like primates, are unable to synthesize AsA due to a hereditary defect in GULO. Neuron numbers in the brain are reduced in the guinea pig, implying a causal connection between the immature neuron development and impaired spatial memory. However, this is not the case for the juvenile AKR1A^−/−^ mice because no apparent difference was observed in the number of neurons, probably due to the recruitment of AsA via cord blood until the time of weaning and high preservation of AsA in brain (Fig. 3).

A deficiency in AsA synthesis is associated with an elevation in oxidative stress in the brains of GNL^−/−^ mice^(9,36,37)^ and GULO^−/−^ mice.^(39)^ While antioxidation is the putative function of AsA, AKR1A^−/−^ mice showed no changes in the levels of redox molecules, such as glutathione and cysteine, in the hippocampus compared to the AKR1A^+/+^ mice (Table 1), which suggests that robust oxidative damage in neuronal cells is not likely the cause for the impaired memory in the juvenile AKR1A^−/−^ mice. Treatment of the young adult AKR1A^−/−^ mice with isoflurane, which has been reported to affect spatial memory formation in juvenile mice by neurodegeneration,^(33,40)^ showed no apparent difference in the spatial memory of the adult AKR1A^−/−^ mice that grew up without AsA supplementation (Fig. 5). This is consistent with anesthetic action of isoflurane, in that it does not affect duration of the loss of the righting reflex of young adult AKR1A^−/−^ mice, while pentobarbital anesthesia delays it compared to AKR1A^+/+^ mice.^(20)^ Given these observations, clinical doses of isoflurane anesthesia would not be expected to cause any severe impairment in spatial memory formation in mice.

AsA reportedly supports the action of dopamine β-hydroxylase, an enzyme that catalyzes the conversion of dopamine to norepinephrine *in vitro*.^(2)^ Adult GULO^−/−^ mice show a mild motor deficit, as observed by their slow swimming in water, which suggests that an AsA deficiency may affect neostriatal pathways via a dopamine-glutamate interaction process.^(41)^ In our study, however, swimming speed was the same in all juvenile mice (Fig. 1C). There are no significant changes in norepinephrine content in adrenal glands between the AKR1A^−/−^ and AKR1A^+/+^ mice.^(21)^ We also observed essentially the same results on neurotransmitters in the hippocampus, as evidenced by the fact that no difference was found in the levels of neurotransmitter amines or acetylcholine in the hippocampus of AKR1A^−/−^ and AKR1A^+/+^ mice (Table 1). We observed slight but significant increase in norepinephrine content in the AKR1A^−/−^ mice with AsA supplementation. AKR1A^−/−^ mice without AsA supplementation also showed a trend of high norepinephrine content. These findings collectively suggest involvement of other enzymatic characteristics of AKR1A than the AsA synthesis in either production or decomposition of norepinephrine in the hippocampus, although actual mechanism is not clear at present. Consistent with our results, it has been reported that an AsA treatment improves spatial memory in APP/PSEN1 mice, a mouse model for Alzheimer’s disease, but does not alter monoamine levels in the nucleus accumbens.^(42)^ In the meantime, AsA appears to play a protective role against the excessive action of glutamate receptor in dopaminergic neurons,^(4)^ which suggests that the modulation of cellular signaling may be involved in spatial memory formation. Thus, precisely how AsA supports spatial memory formation remains unclear, and further studies will be required from the standpoint of the production and function of neuronal transmitters.

A recent study revealed that AKR1A has a novel role in energy metabolism of cells,^(43)^ which may explain not only the function of AsA but also the phenotypic difference of AKR1A^−/−^ mice from GULO^−/−^ mice and GNL^−/−^ mice. AKR1A mice possess *S*-nitroso-coenzyme A reductase activity,^(19)^ which protects sulfhydryl groups in proteins from *S*-nitrosylation, and hence the ablation of AKR1A increases the level of *S*-nitrosylation in proteins. A glycolytic enzyme pyruvate kinase M2 appears to be the main target of *S*-nitrosylation via the *S*-nitroso-coenzyme A, which leads to the suppression of enzymatic activity and causes insufficient ATP production in AKR1A^−/−^ mice.^(43)^ Because neurons are cells in which energy is mainly supplied from glycolysis, neuronal function would be suffered in the case of a short ATP supply. AsA stimulates the release of nitric oxide from the *S*-nitrosylated proteins^(44)^ and, hence, supplemented AsA can cope with *S*-nitrosylation reactions in the absence of AKR1A. Given this action of AsA, it is conceivable that supplemented AsA would protect pyruvate kinase from *S*-nitrosylation-mediated inactivation and rescue neuronal function in AKR1A^−/−^ mice. The influence of a short ATP supply would be evident notably in juvenile mice whose synaptic communication is premature and vulnerable. Thus, there are several possible mechanisms that could cause the impaired spatial memory formation in the juvenile AKR1A^−/−^ mice.

An AsA insufficiency causes a defect in spatial memory formation in juvenile mice but not in young adult mice. Although the status of AsA had no effect on production of neurotransmitters, AsA may support neuronal function either via direct action on neurotransmitter function, ameliorating stress conditions, or maintaining energy metabolism. In any event, these results, point to the importance of taking sufficient amounts of AsA for maintaining proper memory formation particularly at the juvenile stage of animals that are unable to synthesize AsA.

## Figures and Tables

**Table 1:** Neurotransmitters and redox molecules in the hippocampus of mice

Compounds	Amount (nmol/g tissue)		
	WT	KO	KO + AsA
Dopamine	0.257 ± 0.006	0.290 ± 0.038	0.341 ± 0.049
3-Methoxytyramine	4.240 ± 0.355	6.550 ± 1.370	5.620 ± 1.160
3,4-Dihydroxyphenylacetic acid	0.399 ± 0.038	0.568 ± 0.129	0.487 ± 0.125
Homovanillic acid	0.213 ± 0.005	0.248 ± 0.004	0.220 ± 0.036
Norepinephrine	2.530 ± 0.107	2.820 ± 0.019	2.890 ± 0.107*
Normetanephrine	0.237 ± 0.020	0.273 ± 0.065	0.249 ± 0.041
3-Methoxy-4-hydroxyphenylglycol	3.540 ± 0.275	5.050 ± 0.875	4.280 ± 1.150
5-Hydroxytryptamine	3.200 ± 0.074	3.000 ± 0.131	3.130 ± 0.081
5-Hydroxyindoleacetic acid	2.290 ± 0.126	2.720 ± 0.210	2.960 ± 0.473
Glutamic acid	7,780 ± 246.0	7,220 ± 321.0	8,730 ± 690.0
Gamma-aminobutyric acid	932.0 ± 48.70	996.0 ± 63.60	1,660 ± 503.0
Acetylcholine	5.020 ± 0.084	4.540 ± 0.037	6.490 ± 1.430
Choline	130.0 ± 14.70	109.0 ± 8.410	221.0 ± 113.0
Cysteine	62.00 ± 13.30	67.60 ± 9.470	71.79 ± 16.90
Glutathione	1,250 ± 65.90	1,190 ± 61.30	1,570 ± 190.0

Neuroactive amines and related compounds in juvenile mice were measured by HPLC. Other compounds including acetylcholine, glutathione, and amino acids were determined by LC-MS. Data are presented as the mean ± SEM (*n* = 4–5). *p<0.05, indicating a significant difference compared with the WT group.

## References

[1] Mandl J, Szarka A, Bánhegyi G Vitamin C: update on physiology and pharmacology. Br J Pharmacol 2009; 157: 1097–1110. 19508394 10.1111/j.1476-5381.2009.00282.x PMC2743829

[2] Hess CR, McGuirl MM, Klinman JP Mechanism of the insect enzyme, tyramine beta-monooxygenase, reveals differences from the mammalian enzyme, dopamine beta-monooxygenase. J Biol Chem 2008; 283: 3042–3049. 18032384 10.1074/jbc.M705911200

[3] Castro MA, Beltrán FA, Brauchi S, Concha II A metabolic switch in brain: glucose and lactate metabolism modulation by ascorbic acid. J Neurochem 2009; 110: 423–440. 19457103 10.1111/j.1471-4159.2009.06151.x

[4] Ballaz S, Morales I, Rodríguez M, Obeso JA Ascorbate prevents cell death from prolonged exposure to glutamate in an *in vitro* model of human dopaminergic neurons. J Neurosci Res 2013; 91: 1609–1617. 23996657 10.1002/jnr.23276

[5] Guo YE, Suo N, Cui X, Yuan Q, Xie X Vitamin C promotes oligodendrocytes generation and remyelination. Glia 2018; 66: 1302–1316. 29423921 10.1002/glia.23306 PMC6001564

[6] Linster CL, Van Schaftingen E Vitamin C. Biosynthesis, recycling and degradation in mammals. FEBS J 2007; 274: 1–22. 10.1111/j.1742-4658.2006.05607.x 17222174

[7] Nishikimi M, Fukuyama R, Minoshima S, Shimizu N, Yagi K Cloning and chromosomal mapping of the human nonfunctional gene for L-gulono-gamma-lactone oxidase, the enzyme for L-ascorbic acid biosynthesis missing in man. J Biol Chem 1994; 269: 13685–13688. 8175804

[8] Maeda N, Hagiara H, Nakata Y, Hiller S, Wilder J, Reddick R Aortic wall damage in mice unable to synthesize ascorbic acid. Proc Natl Acad Sci U S A 2000; 97: 841–846. 10639167 10.1073/pnas.97.2.841 PMC15418

[9] Kondo Y, Inai Y, Sato Y, et al Senescence marker protein 30 functions as gluconolactonase in L-ascorbic acid biosynthesis, and its knockout mice are prone to scurvy. Proc Natl Acad Sci U S A 2006; 103: 5723–5728. 16585534 10.1073/pnas.0511225103 PMC1458640

[10] Spite M, Baba SP, Ahmed Y, et al Substrate specificity and catalytic efficiency of aldo-keto reductases with phospholipid aldehydes. Biochem J 2007; 405(Pt 1): 95–105. 17381426 10.1042/BJ20061743 PMC1925154

[11] Gabbay KH, Bohren KM, Morello R, Bertin T, Liu J, Vogel P Ascorbate synthesis pathway: dual role of ascorbate in bone homeostasis. J Biol Chem 2010; 285: 19510–19520. 20410296 10.1074/jbc.M110.110247 PMC2885230

[12] Takahashi M, Miyata S, Fujii J, et al *In vivo* role of aldehyde reductase. Biochim Biophys Acta 2012; 1820: 1787–1796. 22820017 10.1016/j.bbagen.2012.07.003

[13] Nishida H, Kurahashi T, Saito Y, et al Kidney fibrosis is independent of the amount of ascorbic acid in mice with unilateral ureteral obstruction. Free Radic Res 2014; 48: 1115–1124. 24735064 10.3109/10715762.2014.915031

[14] Ho HT, Chung SK, Law JW, et al Aldose reductase-deficient mice develop nephrogenic diabetes insipidus. Mol Cell Biol 2000; 20: 5840–5846. 10913167 10.1128/mcb.20.16.5840-5846.2000 PMC86061

[15] Aida K, Ikegishi Y, Chen J, et al Disruption of aldose reductase gene (Akr1b1) causes defect in urinary concentrating ability and divalent cation homeostasis. Biochem Biophys Res Commun 2000; 277: 281–286. 11032718 10.1006/bbrc.2000.3648

[16] Hayashi H, Fujii Y, Watanabe K, Urade Y, Hayaishi O Enzymatic conversion of prostaglandin H2 to prostaglandin F2 alpha by aldehyde reductase from human liver: comparison to the prostaglandin F synthetase from bovine lung. J Biol Chem 1989; 264: 1036–1040. 2910843

[17] Takahashi M, Fujii J, Teshima T, Suzuki K, Shiba T, Taniguchi N Identity of a major 3-deoxyglucosone-reducing enzyme with aldehyde reductase in rat liver established by amino acid sequencing and cDNA expression. Gene 1993; 127: 249–253. 8500767 10.1016/0378-1119(93)90728-l

[18] Kurahashi T, Kwon M, Homma T, et al Reductive detoxification of acrolein as a potential role for aldehyde reductase (AKR1A) in mammals. Biochem Biophys Res Commun 2014; 452: 136–141. 25152401 10.1016/j.bbrc.2014.08.072

[19] Anand P, Hausladen A, Wang YJ, et al Identification of S-nitroso-CoA reductases that regulate protein S-nitrosylation. Proc Natl Acad Sci U S A 2014; 111: 18572–18577. 25512491 10.1073/pnas.1417816112 PMC4284529

[20] Ito J, Otsuki N, Zhang X, et al Ascorbic acid reverses the prolonged anesthetic action of pentobarbital in Akr1a-knockout mice. Life Sci 2014; 95: 1–8. 24355294 10.1016/j.lfs.2013.12.004

[21] Homma T, Akihara R, Okano S, et al Heightened aggressive behavior in mice deficient in aldo-keto reductase 1a (Akr1a). Behav Brain Res 2017; 319: 219–224. 27888021 10.1016/j.bbr.2016.11.038

[22] Vorhees CV, Williams MT Morris water maze: procedures for assessing spatial and related forms of learning and memory. Nat Protoc 2006; 1: 848–858. 17406317 10.1038/nprot.2006.116 PMC2895266

[23] Matsuoka Y, Yamato M, Yamasaki T, Mito F, Yamada K Rapid and convenient detection of ascorbic acid using a fluorescent nitroxide switch. Free Radic Biol Med 2012; 53: 2112–2118. 23026412 10.1016/j.freeradbiomed.2012.09.032

[24] Kobayashi S, Lee J, Takao T, Fujii J Increased ophthalmic acid production is supported by amino acid catabolism under fasting conditions in mice. Biochem Biophys Res Commun 2017; 491: 649–655. 28757411 10.1016/j.bbrc.2017.07.149

[25] Lee J, Kang ES, Kobayashi S, et al The viability of primary hepatocytes is maintained under a low cysteine-glutathione redox state with a marked elevation in ophthalmic acid production. Exp Cell Res 2017; 361: 178–191. 29079265 10.1016/j.yexcr.2017.10.017

[26] Otsuki N, Homma T, Fujiwara H, et al Trichloroethylene exposure aggravates behavioral abnormalities in mice that are deficient in superoxide dismutase. Regul Toxicol Pharmacol 2016; 79: 83–90. 27166294 10.1016/j.yrtph.2016.05.007

[27] Takahashi M, Fujii J, Miyoshi E, Hoshi A, Taniguchi N Elevation of aldose reductase gene expression in rat primary hepatoma and hepatoma cell lines: implication in detoxification of cytotoxic aldehydes. Int J Cancer 1995; 62: 749–754. 7558425 10.1002/ijc.2910620617

[28] Otsu K, Ikeda Y, Fujii J Accumulation of manganese superoxide dismutase under metal-depleted conditions: proposed role for zinc ions in cellular redox balance. Biochem J 2004; 377(Pt 1): 241–248. 14531733 10.1042/BJ20030935 PMC1223854

[29] Fujii T, Endo T, Fujii J, Taniguchi N Differential expression of glutathione reductase and cytosolic glutathione peroxidase, GPX1, in developing rat lungs and kidneys. Free Radic Res 2002; 36: 1041–1049. 12516874 10.1080/1071576021000006725

[30] Kempadoo KA, Mosharov EV, Choi SJ, Sulzer D, Kandel ER Dopamine release from the locus coeruleus to the dorsal hippocampus promotes spatial learning and memory. Proc Natl Acad Sci U S A 2016; 113: 14835–14840. 27930324 10.1073/pnas.1616515114 PMC5187750

[31] Kenney JW, Gould TJ Modulation of hippocampus-dependent learning and synaptic plasticity by nicotine. Mol Neurobiol 2008; 38: 101–121. 18690555 10.1007/s12035-008-8037-9 PMC2683366

[32] Stratmann G, Sall JW, May LD, Loepke AW, Lee MT Beyond anesthetic properties: the effects of isoflurane on brain cell death, neurogenesis, and long-term neurocognitive function. Anesth Analg 2010; 110: 431–437. 10.1213/ANE.0b013e3181af8015 19917621

[33] Yang B, Liang G, Khojasteh S, et al Comparison of neurodegeneration and cognitive impairment in neonatal mice exposed to propofol or isoflurane. PLoS One 2014; 9: e99171. 24932894 10.1371/journal.pone.0099171 PMC4059617

[34] Harrison FE, Green RJ, Dawes SM, May JM Vitamin C distribution and retention in the mouse brain. Brain Res 2010; 1348: 181–186. 20570663 10.1016/j.brainres.2010.05.090 PMC2912448

[35] Kim H, Kim Y, Bae S, et al Vitamin C deficiency causes severe defects in the development of the neonatal cerebellum and in the motor behaviors of Gulo(–/–) mice. Antioxid Redox Signal 2015; 23: 1270–1283. 25977985 10.1089/ars.2014.6043

[36] Son TG, Zou Y, Jung KJ, et al SMP30 deficiency causes increased oxidative stress in brain. Mech Ageing Dev 2006; 127: 451–457. 16500693 10.1016/j.mad.2006.01.005

[37] Sato Y, Kajiyama S, Amano A, et al Hydrogen-rich pure water prevents superoxide formation in brain slices of vitamin C-depleted SMP30/GNL knockout mice. Biochem Biophys Res Commun 2008; 375: 346–350. 18706888 10.1016/j.bbrc.2008.08.020

[38] Tveden-Nyborg P, Johansen LK, Raida Z, Villumsen CK, Larsen JO, Lykkesfeldt J Vitamin C deficiency in early postnatal life impairs spatial memory and reduces the number of hippocampal neurons in guinea pigs. Am J Clin Nut 2009; 90: 540–546. 10.3945/ajcn.2009.27954 19640959

[39] Harrison FE, Meredith ME, Dawes SM, Saskowski JL, May JM Low ascorbic acid and increased oxidative stress in gulo(–/–) mice during development. Brain Res 2010; 1349: 143–152. 20599829 10.1016/j.brainres.2010.06.037 PMC2914834

[40] Liu J, Zhao Y, Yang J, Zhang X, Zhang W, Wang P Neonatal repeated exposure to isoflurane not sevoflurane in mice reversibly impaired spatial cognition at juvenile-age. Neurochem Res 2017; 42: 595–605. 27882447 10.1007/s11064-016-2114-7

[41] Chen Y, Curran CP, Nebert DW, Patel KV, Williams MT, Vorhees CV Effect of vitamin C deficiency during postnatal development on adult behavior: functional phenotype of Gulo^−/−^ knockout mice. Genes Brain Behav 2012; 11: 269–277. 22296218 10.1111/j.1601-183X.2011.00762.x PMC3325330

[42] Kennard JA, Harrison FE Intravenous ascorbate improves spatial memory in middle-aged APP/PSEN1 and wild type mice. Behav Brain Res 2014; 264: 34–42. 24508240 10.1016/j.bbr.2014.01.044 PMC3980584

[43] Zhou HL, Zhang R, Anand P, et al Metabolic reprogramming by the S-nitroso-CoA reductase system protects against kidney injury. Nature 2019; 565: 96–100. 30487609 10.1038/s41586-018-0749-z PMC6318002

[44] Gandley RE, Tyurin VA, Huang W, et al S-nitrosoalbumin-mediated relaxation is enhanced by ascorbate and copper: effects in pregnancy and preeclampsia plasma. Hypertension 2005; 45: 21–27. 15569857 10.1161/01.HYP.0000150158.42620.3e

